# Branchial cleft anomalies: a pictorial review of embryological development and spectrum of imaging findings

**DOI:** 10.1007/s13244-015-0454-5

**Published:** 2015-12-10

**Authors:** Ashok Adams, Kshitij Mankad, Curtis Offiah, Lucy Childs

**Affiliations:** Department of Radiology, Royal London Hospital, Barts Health NHS Trust, London, UK; Department of Radiology, Great Ormond Street Hospital, Great Ormond Street, London, UK

**Keywords:** Branchial arch, Branchial cleft, Pharyngeal apparatus, Pharyngeal pouch, Branchial cleft cyst

## Abstract

**Abstract:**

The branchial arches are the embryological precursors of the face, neck and pharynx. Anomalies of the branchial arches are the second most common congenital lesions of the head and neck in children, with second branchial arch anomalies by far the most common. Clinically, these congenital anomalies may present as cysts, sinus tracts, fistulae or cartilaginous remnants with typical clinical and radiological findings. We review the normal embryological development of the branchial arches and the anatomical structures of the head and neck that derive from each arch. The typical clinical and radiological appearances of both common and uncommon branchial arch abnormalities are discussed with an emphasis on branchial cleft anomalies.

***Key points*:**

• *Anomalies of the branchial arches usually present as cysts, sinuses or fistulae*.

• *Second branchial arch anomalies account for approximately 95 % of cases*.

• *There are no pathognomonic imaging features so diagnosis depends on a high index of suspicion and knowledge of typical locations*.

• *Persistent cysts, fistulae or recurrent localised infection may be due to branchial arch anomalies*.

• *Surgical excision of the cyst or tract is the most common curative option*.

## Introduction

The branchial arches represent the embryological precursors of the face, neck and pharynx. Anomalies of the branchial arches are the second most common congenital lesions of the head and neck in children [1]. They may present as cysts, sinus tracts, fistulae or cartilaginous remnants and present with typical clinical and radiological patterns dependent on which arch is involved. The embryological development of the branchial arches is reviewed and the radiological appearance of branchial arch abnormalities is presented, primarily focusing on branchial cleft anomalies.

## Embryological development of the branchial arches

**Fig. 1:**
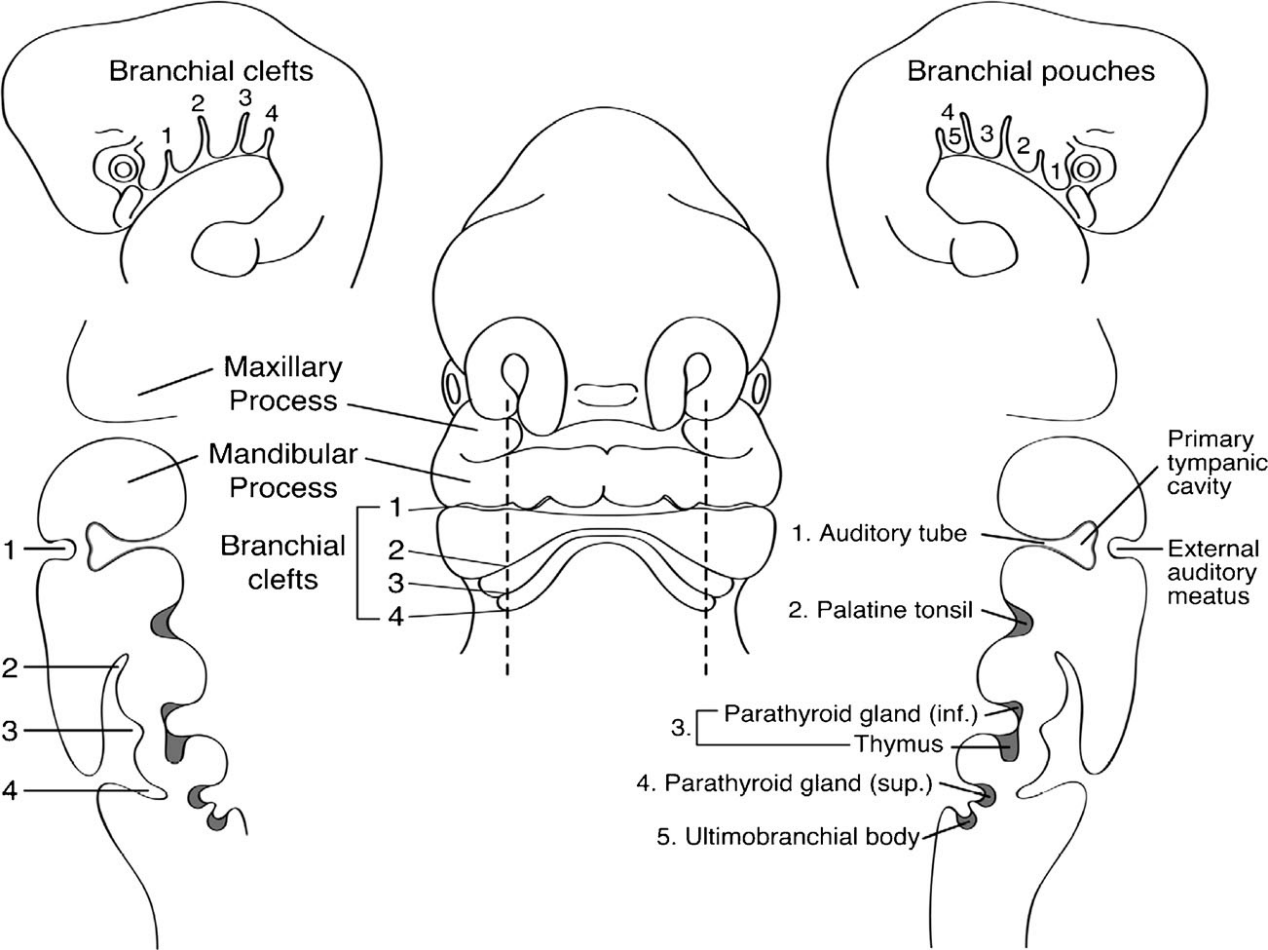
Frontal schematic representation of a 5-mm human embryo at the fifth week of gestation. Sagittal sections taken through the branchial apparatus demonstrate the anatomic relationship of external clefts and internal pouches as well as the derivation of important head and neck structures. The sixth arch is very small and not visualised as a separate, discrete structure from pouch 4/5 in Fig. 1. [Reproduced with permission from Waldhausen J (2006) Branchial cleft and arch anomalies in children. *Seminars in Pediatric Surgery* 15:64–69]

The branchial arches develop between the fourth and seventh week of gestation and form the embryological precursors of the ear and muscles, blood vessels, bones, cartilage and mucosal lining of the face, neck and pharynx (Fig. 1). In total, six pairs of branchial arches form on either side of the pharyngeal foregut in cranio-caudal succession. The fifth pharyngeal arch is usually only rudimentary, or never forms, so ultimately only five arches formulate adult structures [1, 2]. The fifth arch does not contribute to anatomical structures in humans. Schematically, the sixth arch is often represented as part of the fourth arch due to its small size.

Each branchial arch is lined externally by an ectoderm-lined recess, referred to as a pharyngeal/branchial cleft, and internally by a layer of endoderm, referred to as a pharyngeal/branchial pouch (Fig. 1). Between the branchial cleft and branchial pouch is a core of mesenchyme derived from a combination of lateral plate mesoderm, somites and neural crest cells. Clefts and pouches are gradually obliterated by invasion of mesenchyme to form various adult structures (Table 1). Each arch has its own central cartilaginous/skeletal supporting element, an aortic arch artery and an arch-associated cranial nerve. The first arch is supplied by the trigeminal nerve (V), the second arch by the facial nerve (VII), the third arch is supplied by the glossopharyngeal nerve (IX) and the fourth and sixth arches by the superior laryngeal and recurrent laryngeal branches of the vagus nerve (X), respectively [3]. Each nerve innervates structures derived from its associated arch.

**Table 1:** Derivatives of the branchial clefts and pouches. Arch 5 does not form structures in humans and is, therefore, not listed in Table 1

	Pharyngeal arch	Aortic arch artery	Cranial nerve	Muscular structures	Skeletal structures	Adult structures
External auditory meatus	I (Mandibular arch)	Maxillary artery	Trigeminal (V)	Mandibular prominence: muscles of mastication, anterior belly of digastric, tensor tympani, tensor veli palatini, mylohyoid. Maxilliary prominence: none.	Mandibular prominence: mandible, incus, malleus, Meckel’s cartilages. Maxilliary prominence: maxilla, zygomatic bone, squamos temporal bone, palatine bone, vomer.	Middle ear auditory tube, tympanic activity
	II (Hyoid arch)	Stapedial artery, hyoid artery	Facial nerve (VII)	Muscles of facial expression, (buccinator, platysma, auricularis, frontalis, orbicularis oris, orbicularis oculi) stylohyoid, posterior belly of digastric, stapedius.	Lesser horn of the hyoid bone, superior half of hyoid body, stapes, styloid process.	Supratonsillar fossa, crypts of palatine tonsils
Cervical sinus of His	III	Common carotid, internal carotid artery	Glossopharyngeal (IX)	Stylopharyngeus	Greater horn of hyoid bone, inferior half of hyoid body.	Thymus, inferior parathyroid glands
	IV	Right-Proximal subclavian artery Left-Aortic arch	Vagus nerve (X), superior laryngeal nerve	Intrinsic muscles of soft palate, levator veli palatini, cricothyroid	Laryngeal cartilages: thyroid cartilage, cricoid cartilage, arytenoid cartilage, corniculate cartilage, cuneiform cartilage, epiglottic cartilage	Superior parathyroid glands, C-cells of thyroid
	VI	Right – proximal pulmonary artery Left – proximal pulmonary artery and ductus arteriosus	Vagus nerve, recurrent laryngeal nerve	Intrinsic muscles of the larynx, (not cricothyroid)	Laryngeal cartilages: thyroid cartilage, cricoid cartilage, arytenoid cartilage, corniculate cartilage, cuneiform cartilage.	

The pharyngeal cleft associated with the first branchial arch forms the external auditory meatus. The corresponding first pharyngeal pouch gives rise to the tubotympanic recess which forms the tympanic cavity of the middle ear and eustachian tube. The associated cleft between these structures forms the tympanic membrane. The tympanic membrane is, therefore, trilaminar and composed of ectoderm, mesoderm and endoderm [4]. The first branchial arch splits distally to form the mandibular and maxillary processes of the jaw. The muscles of mastication, auditory ossicles (malleus and incus) and multiple bony and cartilaginous facial structures are also derived from the first arch.

The first arch and cranial aspect of the second pharyngeal arch enlarge and thicken combining with the third and fourth pharyngeal arches to form an ectoderm-lined cavity called the cervical sinus of His (or cervical cyst). The prominent ridges around the cavity form various caudal structures in the face and neck that are detailed below. The sinus of His rapidly involutes during embryogenesis. In an adult, the angle between the sternocleidomastoid muscle anteriorly and the infrahyoid strap muscles represents the obliterated sinus [1, 5].

Each of the pharyngeal pouches gives rise to an adult structure. Derivatives of the second pharyngeal arch structures include the tonsillar fossa, palatine tonsils, stapes, lesser horn of the hyoid, adjacent muscles of facial expression and floor of the mouth. The third pharyngeal pouch gives rise to the greater horn of the hyoid, thymus and pyriform fossa. The thymic gland precursors migrate inferiorly and ventral to the thyroid to fuse in the midline, forming a bilobed structure. The inferior parathyroid glands also develop from the third pharyngeal pouch; the superior parathyroid glands, paradoxically, arise more inferiorly from the fourth pharyngeal pouch. The fourth pharyngeal pouch also contributes to the apex of the pyriform fossa, as well as laryngeal cartilages and laryngeal muscles. The ultimobranchial body (ultimopharyngeal body/ultimobranchial gland) is a derivative of the ventral recess of the fourth pharyngeal pouch, technically fifth, but that one is rudimentary and merges with the fourth (Fig. 1). It gives rise to the calcitonin-producing cells (parafollicular cells/C cells) of the thyroid gland. The sixth pharyngeal arch contributes towards the sternocleidomastoid and trapezius muscles.

## Anomalies of the branchial arch apparatus

Branchial arch anomalies are the second most common head and neck congenital lesions in children with thyroglossal duct remnants being the most common. They represent approximately 20 % of cervical masses, hence branchial arch anomalies are considered in the differential diagnosis of head and neck masses in children [6]. Second branchial arch anomalies are the most common and account for approximately 95 % of cases. First branchial arch anomalies account for 1–4 % cases with third and fourth branchial arch anomalies being extremely rare [6]. Branchial arch anomalies may present as cysts, fistulas, sinuses or cartilaginous remnants. Branchial cysts typically present in older children/young adults, whereas fistulas typically present in infants/young children. Branchial arch anomalies may be bilateral in 2–3 % of cases where they are often familial [7]. A sinus is a blind ending tract and in the context of a branchial arch anomaly, may connect with either the skin (branchial cleft sinus) or with the pharynx (branchial pouch sinus). A fistula is a communication between two epithelialised surfaces and with regard to branchial arch anomalies, requires communication between a persistent pouch and cleft. If no communication occurs with the inner mucosa or outer skin, then the trapped branchial arch remnant forms a cyst. There are a number of theories proposed to account for the formation of these anomalies, the most widely accepted theory being that they result from incomplete obliteration of the branchial apparatus, primarily the cleft [8]. In the case of sinuses and fistulae, the pharyngeal membrane and pouch are also implicated.

## Second branchial cleft anomalies

Second branchial cleft anomalies most commonly present as cysts followed by sinuses and fistulae [9]. Most are present within the submandibular space but they can occur anywhere along the course of the second branchial arch tract which extends from the skin overlying the supraclavicular fossa, between the internal and external carotid arteries, to enter the pharynx at the level of the tonsillar fossa [2]. They have previously been classified into four different sub-types by Bailey in 1929 [10]:

- Type I – Most superficial and lies along the anterior surface of sternocleidomastoid deep to the platysma, but not in contact with the carotid sheath.
- Type II – Most common type where the branchial cleft cyst lies anterior to the sternocleidomastoid muscle, posterior to the submandibular gland, adjacent and lateral to the carotid sheath.
- Type III – Extends medially between the bifurcation of the internal and external carotid arteries, lateral to the pharyngeal wall.
- Type IV- Lies deep to the carotid sheath within the pharyngeal mucosal space and opens into the pharynx.

**Fig. 2:**
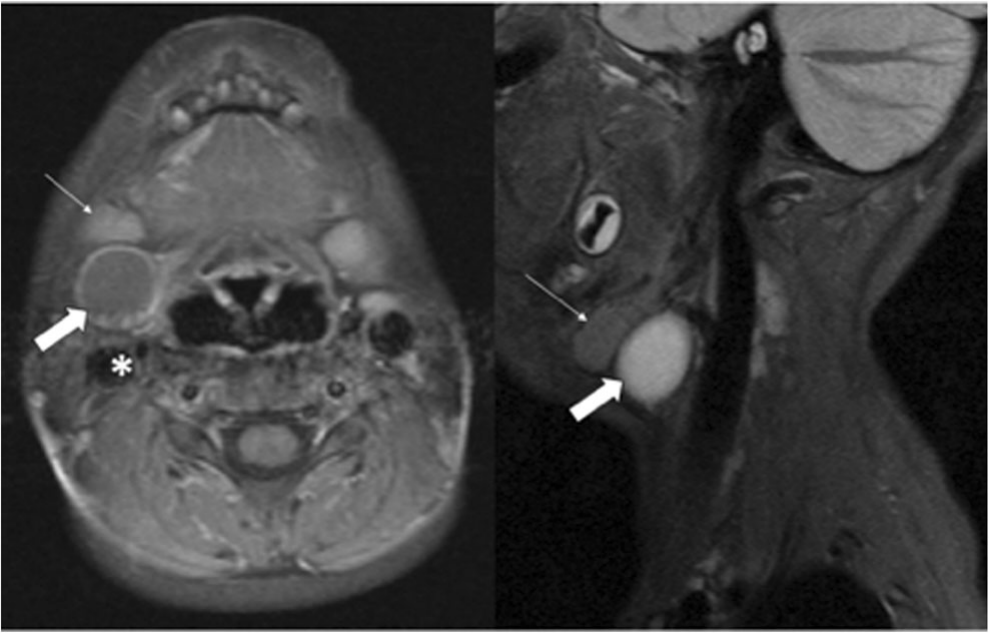
Two-year-old child with axial fat-suppressed T1 post-contrast and sagittal short tau inversion recovery (STIR) images demonstrating a rounded and well-defined T1 isointense, T2 hyperintense lesion with thin peripheral enhancement (*thick white arrows*). It is located posterior to the right submandibular gland (*thin white arrow*) and anterior to the sternocleidomastoid muscle and carotid sheath (*asterisk*). This was confirmed to represent a second branchial cleft cyst following surgical excision

**Fig. 3:**
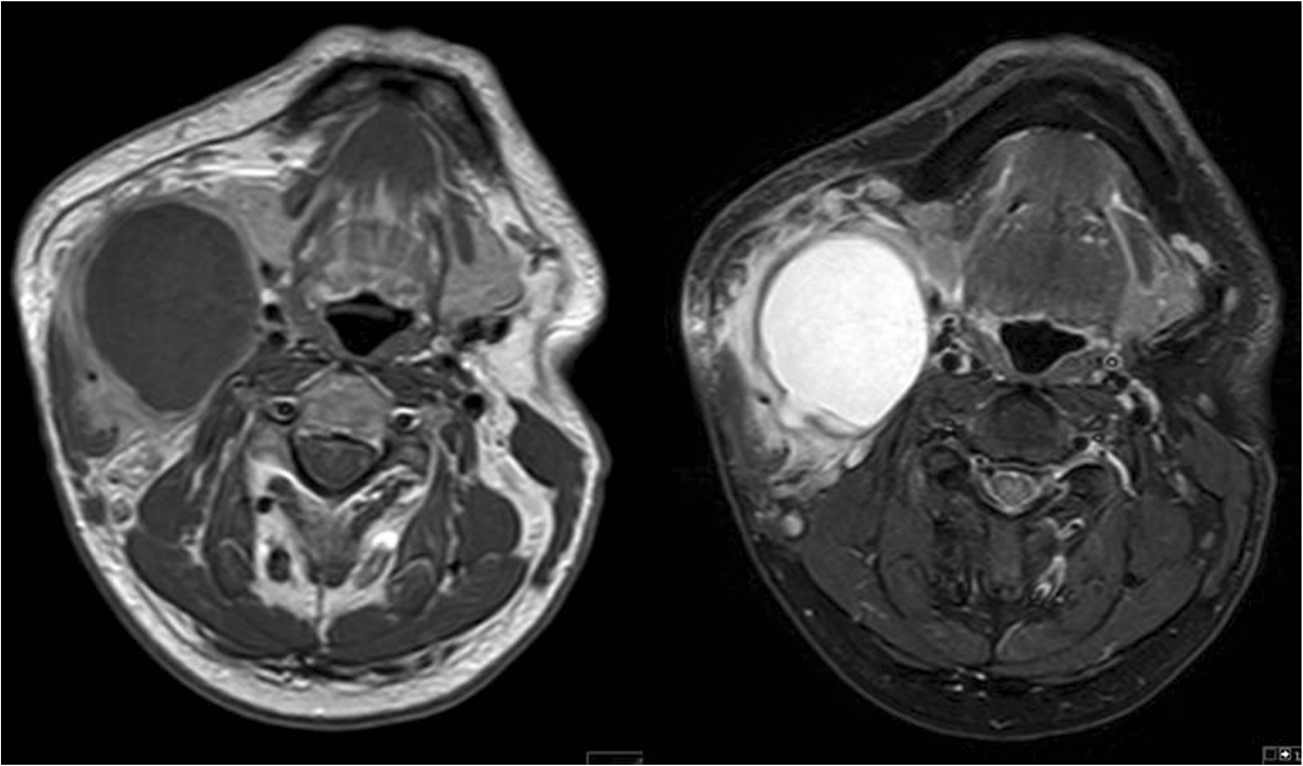
T1 post-contrast and STIR images demonstrate a T2 hyperintense cystic mass with irregular peripheral enhancement after contrast administration. The abnormality was confirmed to be an infected second branchial cleft cyst in a 54-year-old man who had a history of recurrent infections at the mandibular angle

**Fig. 4:**
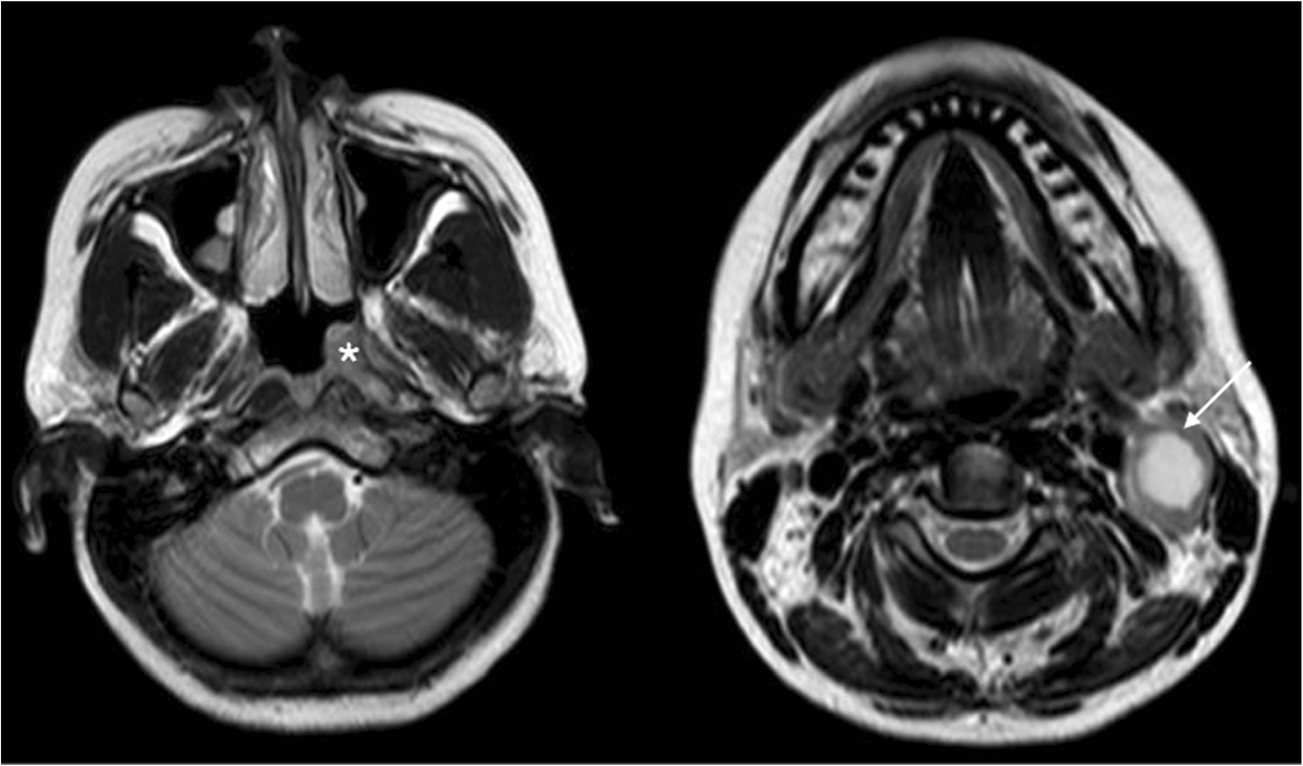
Forty-year-old man presenting with a persistent level II neck lump. Axial T2-weighted imaging demonstrates a complex cystic lesion posterior to the left angle of the mandible (*white arrow*). Asymmetrical soft tissue was also appreciated in the left fossa of Rosenmüller (*asterisk*). A second branchial cleft cyst was considered in the differential, but the diagnosis was finally confirmed to be nasopharyngeal carcinoma with cystic metastases

A fistula or cyst in the lower anterior or lateral region of the neck is most likely to represent a second branchial cleft anomaly (Fig. 2). Fistulae are usually diagnosed in infancy/childhood with drainage of secretions or purulent material from an opening at the anterior border of the sternocleidomastoid within the lower third of the neck. Cysts are most often diagnosed as a painless, compressible lateral neck mass in a child/young adult that may become tender and/or increase in size if they become infected (Fig. 3) [9]. Histologically, they are filled with a turbid yellow fluid containing cholesterol crystals and are lined by stratified squamous epithelium. In adult patients, the main diagnostic consideration is whether the cystic lesion represents a metastatic lymph node and subsequent imaging is directed at identifying a primary neoplastic lesion (Fig. 4). This is particularly true if there is no history of chronic neck fullness and no history of a recurrent mass following upper respiratory tract infections. In the authors’ institution, this is achieved with a combination of cross-sectional imaging [magnetic resonance imaging (MRI) being the preferred modality] and positron emission tomography-computed tomography (PET-CT) prior to pan-endoscopy and tissue biopsy. Occult papillary thyroid cancer is also a recognised cause of cystic metastases and may also seen seen in children. Fluid aspiration in association with thyroglobulin levels may aid the distinction [11].

On ultrasound (US) imaging, second branchial cleft cysts are typically well-circumscribed, thin-walled and anechoic with evidence of compressibility and posterior acoustic enhancement [12]. They may contain internal echoes compatible with internal debris. On CT imaging, they are well-circumscribed, low-density cystic masses with a thin wall [8]. If they become infected, this may become thick-walled with evidence of mural enhancement, localised inflammatory change and peri-lesional fat stranding. The mural thickening is attributed to the response of lymphoid tissue. MRI is better suited in the assessment of deep tissue involvement. On T1-weighted imaging, they may turn from low to high signal depending on the proteinaceous content of the cyst, but are typically hyperintense on T2-weighted imaging, as seen in Fig. 2 [13]. As with CT imaging, mural thickening and enhancement varies with inflammatory change and typically occurs in the setting of infection. A tissue ‘beak’ between the internal and external carotid arteries is pathognomonic of Bailey type III cysts. Surgical management involves complete surgical excision encompassing the external sinus opening with dissection of the sinus tract [14].

## First branchial cleft anomalies

First branchial cleft anomalies can occur anywhere along the course of the first branchial arch tract. This extends from a cutaneous opening in the submandibular triangle, supero-lateral to the hyoid bone, ascending to the region of the parotid salivary gland to terminate at the cartilaginous/bony junction of the external auditory canal. The tract may pass above or below the facial nerve [4].

Work in 1972 subdivided first branchial cleft anomalies into two groups, Type I and Type II, based on presumed pathogenesis with limited applicability from an imaging perspective;

Type I anomalies are purely ectodermal in origin and present as cystic masses adjacent to the external auditory canal. They are duplications of the membranous external auditory canal and contain squamous epithelium but no skin adnexa or cartilage.

Type II anomalies may present as cysts, sinuses or fistulae in the region of the angle of the mandible. They are ectodermal and mesodermal in origin, hence they contain squamous epithelium, skin adnexa (hair follicles, sweat and sebaceous glands) and cartilage.

**Fig. 5:**
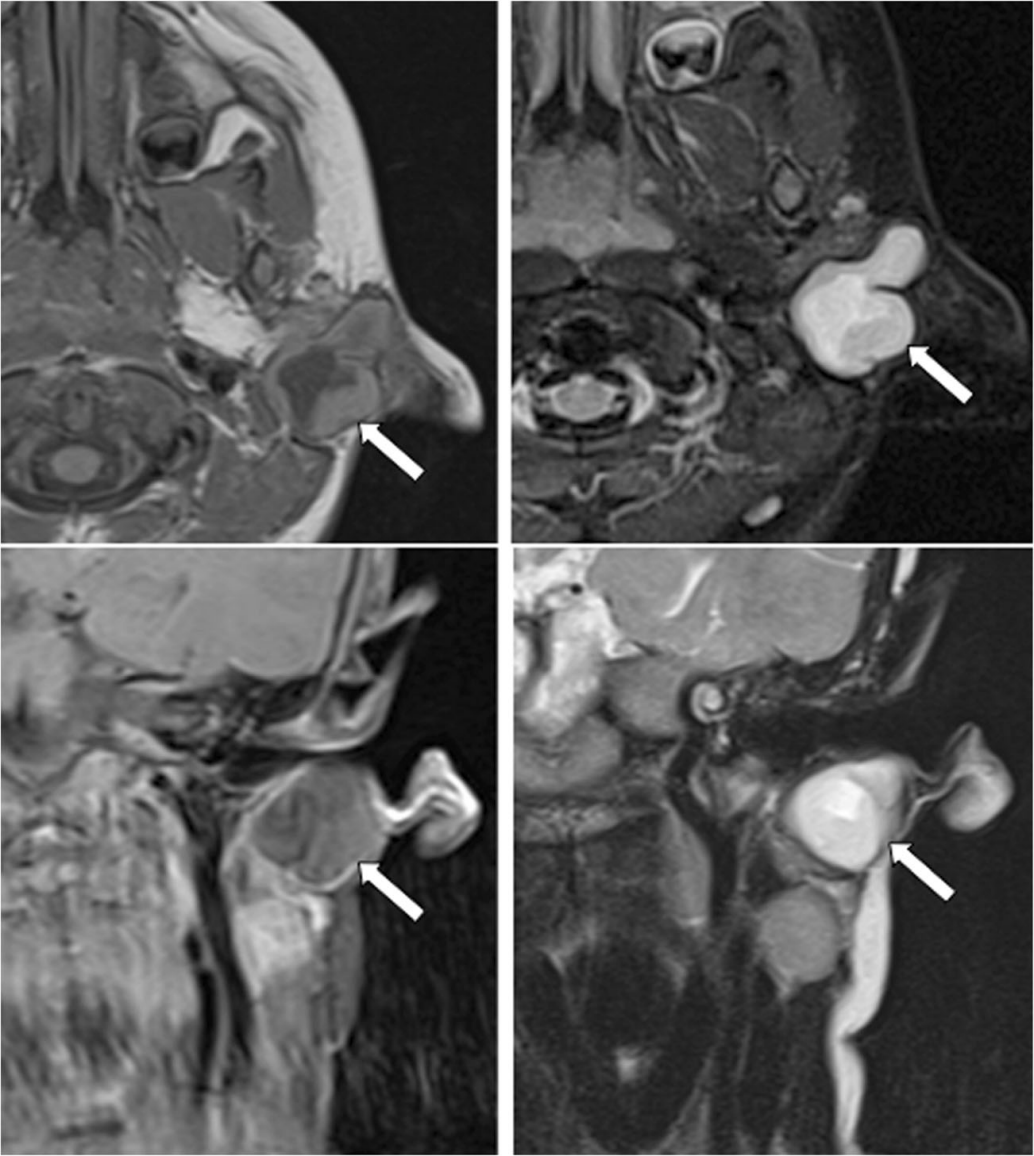
MR imaging from a child presenting with purulent ear discharge. Axial and coronal T1 post-contrast (*left hand images*) with axial and coronal STIR images (*right hand images*) demonstrating a thick-walled sinus tract (*white arrows*) that extended to the clinically apparent opening in the left external auditory canal. The tract was surgically excised and confirmed to represent a first branchial cleft anomaly

**Fig. 6:**
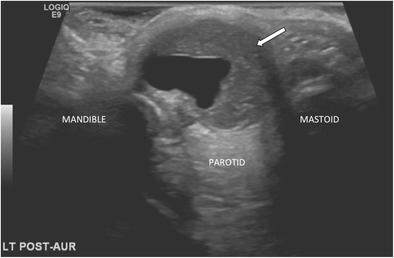
Transverse ultrasound (US) image from the same child as in Fig. 5, confirming the presence of a thick-walled cystic structure that was part of the sinus tract that extended to the left external auditory canal (*white arrow*)

First branchial cleft anomalies are often misdiagnosed and often inadequately treated before they are surgically excised. They are more common in females and typically present as cyst/sinus/fistula between the external auditory canal and submandibular area. They may present with cervical symptoms and drainage from a pit-like depression at the angle of the mandible. If infected, the drainage may be purulent and there may be associated adenitis in the submandibular area. First branchial cleft anomalies can also present with an inflammatory mass in the parotid region or present with auricular symptoms and drainage of mucus or pus from the external auditory canal (Figs. 5 and 6) [15].

On CT imaging, first branchial cleft anomalies usually present as a cystic mass superficial/within/deep to the parotid salivary gland [16]. As with other branchial cleft anomalies, cyst wall thickness and enhancement vary with the degree of inflammation.

**Fig. 7:**
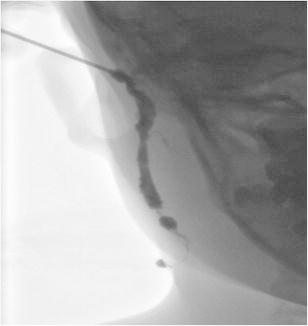
A sinogram performed on a child prior to surgical excision for a presumed first branchial cleft fistula. The opening within the right external auditory canal was cannulated and water-soluble contrast media injected confirmed the presence of a fistulous tract. During the procedure, contrast media was noted to pass via the tract through an external cutaneous opening in the right submandibular region

First branchial cleft anomalies, as with other branchial cleft anomalies, typically do not regress spontaneously and have a propensity for recurrent infections; hence, surgical excision is advised for definitive treatment [14]. This involves complete resection with preservation of normal structures and utilises a superficial parotidectomy approach with potential risk of injury to the facial nerve. The extent of the anomaly is typically underestimated and a significant cutaneous tract may be present and necessitate excision of involved skin and cartilage of the external auditory canal. Pre-operatively, a sinogram may be performed in order to delineate the extent of the tract, although this is not routinely performed in our institution (Fig. 7).

## Third and fourth branchial cleft anomalies

Third and fourth branchial cleft anomalies may appear similar to second branchial cleft anomalies externally with a cutaneous opening in the supraclavicular area; however, internally, they enter the pharynx through the pyriform sinus below the hyoid bone. Third and fourth branchial cleft anomalies are distinguished anatomically by their relationship to the superior laryngeal nerve with third pharyngeal cleft anomalies above and fourth pharyngeal cleft anomalies below. The internal opening helps to determine their origin and a third branchial cleft sinus arises from the rostral end of the pyriform fossa [2]. Third branchial cleft fistulae/sinus tracts typically present earlier than third branchial cleft cysts. Most third branchial cleft cysts present in the posterior cervical space, posterior to the sternocleidomastoid muscle as a painless, fluctuant mass that may enlarge and become tender if infected. An infected third branchial cleft cyst should be considered if a patient presents with an abscess in the posterior triangle of the neck.

**Fig. 8:**
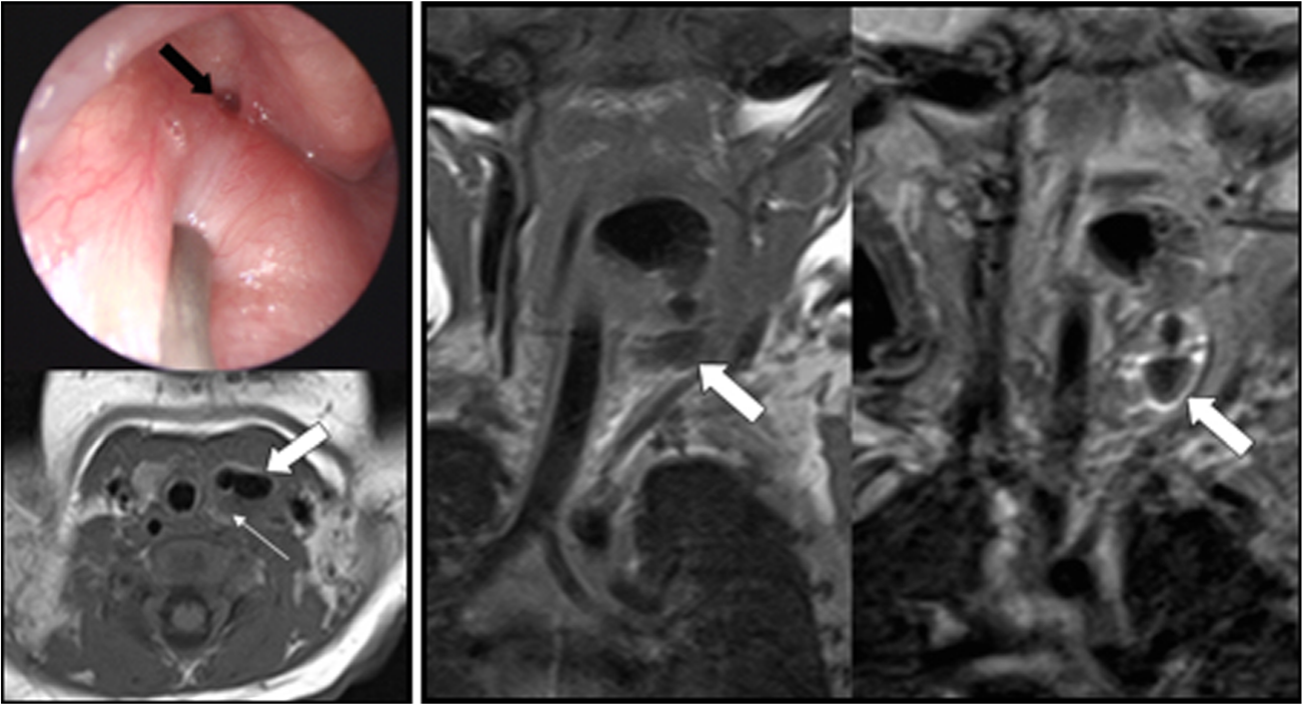
This 18-day-old baby presented with intermittent stridor. On examination, a left-sided oropharyngeal swelling was identified. The baby had initially required continuous positive airway pressure. Microlaryngobronchoscopy identified an internal opening arising from the pyriform sinus apex (*black arrow*; image courtesy of Mr Y. Bajaj). Axial and coronal T1 with coronal STIR imaging identified a likely air-containing structure (*thick white arrows*) extending from the pyriform sinus to the level of the left thyroid gland that was abnormally small (*thin white arrow*). This was surgically confirmed to represent a fourth branchial cleft sinus tract

A fourth branchial cleft fistula/sinus tract arises from the pyriform sinus apex and descends inferiorly to the mediastinum in the path of the tracheo-oesophageal groove [13, 17]. They are commonly left-sided for reasons that are yet to be established and most often present as a sinus tract coursing from the apex of the pyriform fossa to the upper aspect of the left thyroid lobe (Fig. 8). As a result, patients can present with a recurrent abscess in the low anterior neck and/or recurrent suppurative thyroiditis. It can be difficult to determine the exact tract on imaging and this can be achieved with a barium swallow test that can also be combined with a CT neck study after the barium swallow. Due to the anatomical course, complete resection with open neck surgery is difficult and new endoscopic techniques that involve treatment with cauterisation have been developed with success [14].

There are no pathognomonic clinical or imaging features of branchial arch anomalies; therefore, diagnosis is dependent upon the radiologist having a sound knowledge of the types of branchial arch malformations and their typical location. The diagnosis should be considered when persistent cysts or fistulae are present in the head and neck and correlate this with appropriate clinical history and examination. Differential diagnoses for cysts and sinus tracts in the head and neck should be considered (Table 2).

**Table 2:** Branchial arch anomalies and their associated differential diagnoses

Cleft	Differential diagnoses
First branchial cleft anomaly	Parotitis with abscess formation lymphatic malformation parotid sialocoele benign lymphoepithelial cyst
Second branchial cleft anomaly	Lymphatic malformation cystic nodal metastases lymphadenopathy/abscess schwannoma
Third branchial cleft anomaly	Lymphatic malformation abscess cervical thymic cyst infrahyoid thyroglossal duct cyst cystic nodal metastases laryngocoele
Fourth branchial cleft anomaly	Cervical thymic cyst lymphatic malformation thyroglossal duct cyst thyroid colloid cyst abscess

## Conclusion

The branchial arches form embryological precursors of tissues within the head and neck. Branchial arch anomalies represent 20 % of cervical neck masses in children and typically result from incomplete obliteration of branchial clefts with subsequent formation of cysts, fistulae and sinus tracts. Second branchial cleft anomalies represent the most frequent subtype with surgical excision being the most common curative option. The location, clinical symptoms, imaging findings and a high index of suspicion aid the diagnosis of this relatively common diagnosis.
